# Comparison of live birth rates between calorie-restricted diet and metformin prior to ovulation induction therapy in women with polycystic ovary syndrome and overweight/obesity: a protocol for a multicenter, randomized comparative effectiveness trial

**DOI:** 10.3389/fpubh.2026.1881719

**Published:** 2026-09-10

**Authors:** Yingying Lu, Junjie Qu, Yingying Yang, Jiani Sun, Lulu Geng, Ling Hong, Lisa Moran, Helena Teede, Manna Zhang, Lihua Wang, Miaoxin Chen

**Affiliations:** 1 Shanghai Key Laboratory of Maternal Fetal Medicine, Shanghai Institute of Maternal-Fetal Medicine and Gynecologic Oncology, Shanghai First Maternity and Infant Hospital, School of Medicine, Tongji University, Shanghai, China; 2 Reproductive Medical Center, Lianyungang Maternal and Child Health Hospital, Lianyungang, Jiangsu, China; 3 Monash Centre for Health Research and Implementation (MCHRI), Monash University, Melbourne, VIC, Australia; 4 Department of General Practice, Shanghai Tenth People’s Hospital, School of Medicine, Tongji University, Shanghai, China; 5 Shanghai Jiao Tong University School of Medicine Affiliated Renji Hospital, Shanghai, China

**Keywords:** calorie-restricted diet, live birth, overweight and obesity, polycystic ovary syndrome, randomized comparative effectiveness trial

## Abstract

**Background:**

Polycystic ovary syndrome (PCOS) significantly impairs physical and mental health, quality of life, and fertility in women of reproductive age. Strategies to enhance fertility in PCOS have been prioritised in International Guidelines. Whilst Guidelines recommend Letrozole in first line ovulation induction (OI), therapeutic interventions to optimise endocrine and metabolic PCOS features prior to OI have not been adequately researched despite potential to improve live birth and pregnancy outcomes. Whilst both a calorie-restricted (CR) diet and metformin interventions may offer benefits in the context, evidence is limited including for live birth rates, and pregnancy outcomes. Therefore, this study will aim aims to compare the effects of a CR diet versus metformin on live birth, endocrine, reproductive, and metabolic parameters as well as pregnancy outcomes in women with PCOS and overweight/obesity.

**Method:**

This multicenter, randomized (1:1) comparative effectiveness trial will evaluate and compare the effects of a CR diet versus metformin, administered for 12 weeks prior to OI, on live birth, endocrine, metabolic, reproductive, and pregnancy outcomes in women with PCOS and overweight/obesity. The primary analysis will follow the intention-to-treat principle, including all participants who underwent randomization.

**Discussion:**

This trial investigates the comparative efficacy of a CR diet versus metformin over a 12-week period prior to OI therapy in women with PCOS and overweight/obesity. Early therapeutic intervention holds significant potential to disrupt the pathophysiological cycle characterized by hyperandrogenism, excessive adiposity, and insulin resistance for improving fertility and pregnancy outcomes.

**Clinical trial registration:**

ClinicalTrials.gov, identifier NCT06049186.

## Introduction

Polycystic ovary syndrome (PCOS), a prevalent endocrine and reproductive disorder, significantly impairs the physical and mental health as well as the quality of life in women of reproductive age, with a prevalence reaching up to 12% (1–3). Notably, the global burden of PCOS is escalating at an alarming rate, establishing it as a significant public health challenge (4). PCOS is an endocrine condition with increased insulin resistance, hyperandrogenism, and elevated anti-Müllerian hormone levels. Key clinical manifestations include reproductive (menstrual irregularities, infertility, and high risk pregnancy) (5), metabolic (overweight/obesity, diabetes, and cardiovascular disease) (6–8) alongside psychological and dermatological features (2). These factors may independently or synergistically contribute to diminished fertility in women with PCOS, recognized as the leading cause of anovulation infertility, where infertility is clinically defined as the inability to achieve a clinical pregnancy after 1 year or more of regular, unprotected sexual intercourse (8). In recent years, strategies to enhance fertility in women with PCOS have garnered substantial attention and have become a critical focus in clinical research and practice.

The pathogenesis of PCOS remains incompletely elucidated, and genetic factors and environmental factors such as lifestyle and obesity have been implicated in its aetiology (9). PCOS is a markedly heterogeneous disorder, characterized by complex and variable clinical manifestations (10). It is frequently associated with significant metabolic abnormalities, a subset of which appear to be intrinsic to the syndrome yet are further exacerbated by excess adiposity, which also contributes to its increased prevalence (3, 11). Beyond its endocrine and metabolic manifestations, PCOS is associated with anxiety, depression, and eating disorders (12, 13). Therefore, PCOS should be conceptualized as both a metabolic, psychological and reproductive disorder.

Metformin, a well-established insulin-sensitizing agent, is extensively utilized in the management of endocrine and metabolic features in women with PCOS and overweight/obesity (14). In women with PCOS and obesity, metformin therapy has been demonstrated to improve insulin sensitivity, attenuate hyperinsulinaemia and lower circulating androgen concentrations, and improve menstrual regularity and ovulation rates (15–19). However, conflicting evidence regarding the efficacy of metformin has been reported, which may be attributed to heterogeneous treatment responses among specific patient subgroups (20, 21). In women with PCOS, the clinical limitations of metformin use are primarily related to gastrointestinal adverse effects, such as nausea, vomiting, abdominal pain, and diarrhea (15, 22). Long-term therapy has also been associated with vitamin B12 deficiency (15, 22). Approximately 5% of patients demonstrate intolerance to metformin therapy (23). These limitations highlight the need to develop alternative therapeutic strategies that offer improved safety and efficacy for managing PCOS and its associated metabolic complications.

Current international evidence-based guidelines for PCOS management strongly emphasize the critical role of dietary interventions in women with PCOS and overweight/obesity (14, 24). Dietary interventions have been demonstrated to improve endocrine, metabolic and psychological parameters in women with PCOS (25, 26), with the majority of population studies primarily focusing on weight-related indices, glycolipid metabolism, and hormonal profiles (27, 28). However, evidence concerning their effects on definitive reproductive endpoints, including pregnancy and live birth rates, remains limited (27, 28). Notably, the few existing dietary intervention trials suggest potential benefits in increasing pregnancy and ovulation rates, underscoring the need for further rigorous research to elucidate their impact on clinical fertility outcomes (27). Effective dietary management of PCOS necessitates a multidisciplinary approach encompassing dietitians, endocrinologists, reproductive specialists, and other healthcare professionals (29–31). Such interdisciplinary collaboration is crucial for addressing not only weight reduction but also endocrine and metabolic dysfunctions, as well as reproductive complications (29–31). Beyond these goals, this team-based strategy additionally aims to support psychological well-being, enhance patient motivation, and mitigate the pervasive adverse effects of weight stigma on both clinical care and patient self-perception (29–31). In parallel with lifestyle management, pharmacological options for obesity have expanded in recent years, particularly with the introduction of glucagon-like peptide-1 receptor agonists (32). These agents can produce substantial weight loss and improve metabolic parameters, and emerging evidence suggests potential benefits in women with PCOS and overweight/obesity (33). However, their use in women actively planning pregnancy is limited by considerations regarding use around conception and the need for treatment discontinuation before pregnancy. Therefore, effective non-pharmacological strategies for preconception metabolic management remain clinically important.

A calorie-restricted (CR) diet, as an effective strategy for weight loss and prevention of chronic metabolic diseases, may represent a promising non-pharmacological and non-surgical intervention for women with PCOS and overweight/obesity (34). Several studies have demonstrated that CR diets reduce abdominal adiposity and enhances insulin sensitivity. Furthermore, CR diets are associated with elevated serum sex hormone-binding globulin (SHBG) levels and decreased free androgen index (FAI) and testosterone (T) concentrations (35, 36). However, most previous studies have focused on metabolic outcomes or investigated dietary intervention in combination with metformin, and direct comparative evidence between CR diet and metformin remains lacking (37). Metformin was selected as the active comparator because it is a widely used insulin-sensitizing pharmacological intervention in women with PCOS and overweight/obesity, whereas CR diet represents a non-pharmacological approach that may target overlapping metabolic and endocrine abnormalities through energy restriction and weight loss. Comparing these two approaches before ovulation induction (OI) therefore provides a clinically relevant opportunity to determine whether metabolic optimization through CR diet can translate into improved reproductive outcomes relative to metformin. It remains unclear whether a CR diet can improve definitive reproductive outcomes, particularly live birth, compared with metformin when administered before OI. Recent evidence further suggests that individual reproductive characteristics, including age, BMI, and AMH, may contribute to variability in reproductive prognosis among women with PCOS undergoing fertility treatment (38, 39). Therefore, the primary objective of this trial is to determine whether a 12-week CR diet administered before OI is superior to metformin in improving the live birth rate in women with PCOS and overweight/obesity. We hypothesize that the CR diet will result in a higher live birth rate than metformin. The trial will additionally evaluate whether CR diet may provide an effective non-pharmacological strategy for metabolic and reproductive optimization before OI.

## Methods

### Study design

**Figure 1:**
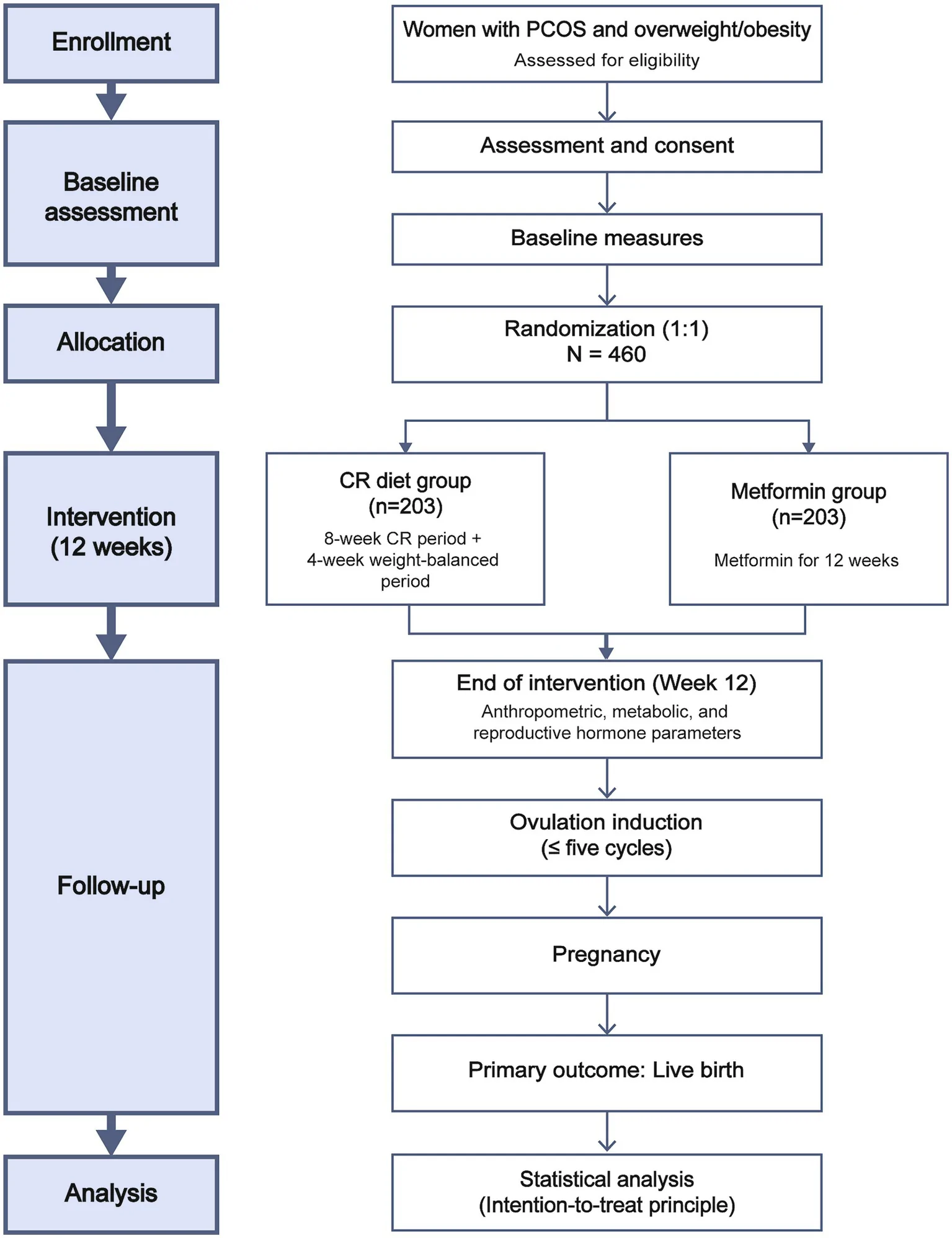
Study flow chart. PCOS, polycystic ovary syndrome; CR, calorie-restricted.

This multicenter, randomized (1:1) comparative effectiveness trial will be conducted to evaluate and compare metabolic and reproductive outcomes between a CR diet and metformin over a 12-week period prior to OI therapy in women with PCOS and overweight/obesity from numerous hospitals nationwide in China. Female participants meeting the predefined eligibility criteria will be recruited, after which eligible subjects will be randomly assigned to either the CR diet group or the metformin intervention group. Following the intervention, standard OI therapy will be administered to participants in both study groups, and subsequent pregnancy outcomes will be monitored. The study flow chart is illustrated in Figure 1. The trial protocol has been designed and is reported according to the Standard Protocol Items: Recommendations for Interventional Trials (SPIRIT) (see Supplementary Material), and the trial results will be reported in accordance with the Consolidated Standards of Reporting Trials (CONSORT). The study protocol has received ethical approval from the Ethics Committee of Shanghai First Maternity and Infant Hospital (Approval Number: KS23168). All procedures involving human participants are in accordance with the ethical standards of the institutional research committee. Written informed consent will be obtained from all participants prior to enrollment. This study was registered at ClinicalTrials.gov (NCT06049186) on 14 September 2023.

### Aims of this study

#### Primary

To determine whether a 12-week CR diet administered prior to OI is superior to metformin in improving the live birth rate among women with PCOS and overweight/obesity. We hypothesize that the CR diet will result in a higher live birth rate than metformin.

#### Secondary

To compare the effects of a CR diet and metformin on secondary reproductive outcomes and changes in anthropometric, metabolic, and reproductive hormone parameters prior to OI in women with PCOS and overweight/obesity. Key secondary outcomes include pregnancy-related outcomes, body weight, glucose and insulin-related measures, lipid profiles, and reproductive hormone levels.

### Eligibility criteria

Inclusion criteria: (1) Women aged between 20 and 35 years, (2) body mass index (BMI) ≥ 23 kg/m^2^ based on the WHO Asian population criteria (40), (3) diagnosed with PCOS based on the Rotterdam diagnostic criteria, as updated in the 2023 International Evidence-Based Guideline for the Assessment and Management of PCOS (14). PCOS was diagnosed when at least two of the following three features were present: (a) oligo- or anovulation, (b) clinical and/or biochemical hyperandrogenism, and (c) polycystic ovarian morphology (PCOM) on ultrasound.

Exclusion criteria: (1) pre-existing type 2 diabetes mellitus, (2) uncontrolled thyroid disorders, (3) acute or chronic infections, (4) severe cerebrovascular or cardiovascular diseases or organic heart disease, (5) history of malignant tumors, (6) use of glucocorticoids or anti-androgen drugs (e.g., spironolactone, cyproterone acetate, flutamide, oral contraceptive pills) within the past 3 months, (7) congenital or acquired uterine anomalies, (8) prior bariatric surgery, (9) currently undergoing weight loss treatment and having experienced weight loss > 5% within the past 3 months, (10) severe hepatic or renal dysfunction, (11) tubal obstruction, (12) total number of motile sperm in male partner < 10 million.

### Sample size

According to the literature (41, 42), live birth rates among women with PCOS in the control arm were around 31.0%. Based on findings from other studies in fertility care and expert opinions from gynecologists and epidemiologists, we assume that a minimal clinically important difference to make CR preferable over conventional diet would be 15.0% (43). To detect this difference using a two-sided test with a 5.0% alpha error and 80% statistical power, and accounting for a potential dropout rate of 20% (44), the minimum numbers of participants required for the study will be 406. Participants will be randomized in a 1:1 ratio between the intervention and control groups. No interim analysis or sample size re-estimation is planned, and the prespecified sample size will be maintained throughout the trial. As the true intervention effect may differ from that assumed in the sample size calculation, the final treatment effect will be reported with its corresponding 95% confidence interval to indicate the magnitude and precision of the observed effect.

### Recruitment and randomization

Potential participants will be recruited from hospital outpatient clinics across multiple centers. Individuals interested in participating will be provided with detailed information about the study protocol, followed by a discussion of study procedures and a screening visit about health and medical history based on inclusion and exclusion criteria via telephone or web. Potential participants will be informed that they will be randomly assigned to either the CR diet group or the metformin group, and that they will not be able to choose their group. Individuals who meet any of the exclusion criteria will be deemed ineligible. Eligible participants will be required to provide written informed consent and complete all baseline assessments before randomization. Block randomization will be performed using a computer-generated random number sequence (command RALLO in SAS) with a fixed block size of 10 to ensure balanced allocation between the two groups across centers. Group assignments will be prepared in sealed, opaque envelopes by a team member not involved in participant recruitment or assessment to ensure allocation concealment. The envelopes will not be revealed to participants until the completion of baseline data collection. Serum and fecal sample will be collected and stored for future use in ancillary studies. Given the nature of the interventions (a CR diet versus metformin), blinding of participants and personnel delivering the interventions is not feasible. However, outcome assessors and data analysts will be blinded to treatment allocation. Outcome assessors will not have access to participants’ treatment allocation information during outcome ascertainment. For statistical analyses, treatment groups will be coded using non-identifying group labels, and data analysts will remain blinded to the corresponding intervention assignments until the prespecified primary analyses have been completed. These procedures are intended to minimize potential assessment and analytical bias. The research protocol, V1.0, was completed on 1 April 2023.

### Intervention

#### Overview

After completing baseline questionnaires, scales, and anthropometric measurements with the assistance of field investigators and dietitians, eligible subjects will be randomly assigned to either the CR diet group or the metformin group in a 1:1 ratio. Participants randomized to the CR diet group will receive standard dietary management, which includes a target energy intake assessment, a pre-intervention education session, and an app-based CR diet program consisting of an 8-week CR phase followed by a 4-week weight maintenance phase. Participants randomized to the metformin group will receive enteric-coated metformin tablets at a total daily dose of 1,500 mg. All participants will be instructed not to alter their physical activity habits throughout the trial to minimize potential confounding factors (45, 46). After the 12-week intervention period, both interventions (CR diet and metformin) will be discontinued, and all participants will undergo OI therapy (up to 5 cycles).

#### CR diet group

To implement the CR diet, the total energy expenditure (TEE) of each participant will be calculated, and the target daily energy intake will be set at 30–50% below the baseline TEE, depending on the participant’s BMI (47). Before the intervention, the International Physical Activity Questionnaire Short Form (IPAQ-SF), along with weight, height, and age, is used to estimate TEE. The model for calculating TEE is based on basal metabolic rate (BMR) and physical activity level (PAL), as recommended by FAO/WHO/UNU Expert Consultation Report (48, 49):

TEE=BMR∗PAL (1)

In Equation 1, BMR is calculated using the Mifflin-St Jeor equation (50), which incorporates weight, height, and age, as shown in Equation 2:

$$\begin{array}{c} \mathrm{BMR}=[9.99\times \text{Weight}\;(\mathrm{kg})]+[6.25\times \text{Height}\;(\mathrm{cm})]\\ -[4.92\times \mathrm{Age}\;(\text{years})-161] \end{array}$$ (2)

The PAL value in Equation 1 is determined using IPAQ-SF, which categorizes individuals into activity levels, such as low-level physical activity, moderate-level physical activity, and high-level physical activity (51, 52). The activity levels are then assigned a corresponding PAL, for example, PAL = 1.4 for low-level physical activity, PAL = 1.6 for moderate-level physical activity, and PAL = 1.8 for high-level physical activity (49, 53).

Before the intervention, participants will receive individualized diet plans based on their calculated target energy intake and will be educated on the principles of reducing daily caloric intake. The prescribed macronutrient distribution for each participant will allocate 40–55% of total energy intake to carbohydrates, 15–20% to protein, and 20–30% to fat, aligning with the ranges recommended by current dietary guidelines (54). Participants may select foods according to their usual dietary habits and preferences while adhering to the prescribed energy and macronutrient targets. During dietary counseling, dietitians will consider regional dietary practices, food availability, and affordability to improve the feasibility and adherence to the CR diet. Participants will upload dietary records, and feedback will be provided on whether their macronutrient intake falls within the target ranges to help them maintain the prescribed distribution. In addition, participants will be instructed not to alter their physical activity habits throughout the trial to minimize potential confounding.

To enhance adherence to the diet intervention in the CR diet group, a custom healthcare service application (app) will be utilized. The app includes two components: a mobile app for participants and an administrator interface for researchers. Through the mobile platform, participants can record food diaries, communicate with dietitians, and receive messages. The dietitians and investigators can access all submitted information and interact with participants via the administrator interface.

Participants will be instructed to record their daily food and beverage consumption using household measures via the app during the 12 weeks of the intervention. Daily uploads including household measurements, photographs, and detailed food descriptions will primarily be used to support dietary adherence through self-monitoring and to provide individualized feedback on the macronutrient distribution. For an objective assessment of actual dietary intake, three 24-h dietary recalls (covering two weekdays and one weekend day) will be extracted by dietitians from participants’ uploaded food diary records. Daily dietary intake will be calculated based on the reported food types and portion sizes, using the nutrient composition data from the Chinese Food Composition Table (55). Adherence to the CR diet will be quantitatively assessed using app-based dietary records through two complementary measures: recording adherence and CR-target adherence. Recording adherence will be calculated as the number of weeks in which a participant records dietary intake on at least 3 days divided by the 12-week intervention period, multiplied by 100%. CR-target adherence will be calculated as the number of weeks in which the participant achieves the prescribed calorie-restriction target divided by the number of weeks meeting the dietary recording criterion, multiplied by 100%. For both measures, adherence will be categorized as high (≥80%), moderate (60–79%), or low (<60%).

During the first 8 weeks of the intervention, dietitians will provide individualized feedback to each participant twice a week via the app. Feedback will be informed by quantified dietary intake, comprising total energy and the macronutrient distribution (carbohydrates, protein, and fat), derived from participants’ uploaded food-diary records (55). Tailored recommendations regarding adjustments to energy intake and food choices will be subsequently issued by dietitians to ensure adherence to the CR protocol. During the subsequent 4-week weight-maintenance period, dietitians will discontinue active dietary intervention and no longer provided feedback on the CR protocol. However, participants will be required to continue recording their food diaries through the app.

#### Metformin group

After randomization, participants assigned to the metformin group will initially receive 1,000 mg per day (500 mg administered twice daily) for the first 3 days to enhance drug tolerability. Thereafter, the dosage will be escalated to the target level of 1,500 mg per day (500 mg administered three times daily) and maintained for the remainder of the 12-week intervention period. Although standard clinical practice often starts metformin at 500 mg/day and increases the dose weekly to minimize gastrointestinal side effects, this rapid escalation regimen was chosen based on prior clinical experience in this population. Participants will be closely monitored for gastrointestinal adverse effects, and dose adjustments or discontinuation will be considered when clinically necessary. They will receive routine clinical advice regarding dietary habits according to standard care and will not receive any materials or interventions related to the CR diet. However, no formal dietary measurements will be conducted during the 12 weeks of the intervention.

#### OI treatment

Prior to initiating OI therapy, a β-human chorionic gonadotropin (β-hCG) test must be performed to exclude pregnancy. Letrozole administration will begin on days 3–5 of the menstrual cycle, with an initial dosage of 5 mg/day. After five consecutive days of administration, transvaginal ultrasonography will be used to monitor follicular development. Based on ultrasonographic findings, human menopausal gonadotropin (HMG) 75 IU may be administered intramuscularly on a daily basis, with subsequent dosage adjustments determined by the follicular response. When the dominant follicle reaches a diameter of ≥14 mm, the monitoring frequency should be increased to alternate-day assessments, supplemented by endocrine profiling, and urinary luteinizing hormone (LH) surge detection. Once follicular maturation criteria are met (i.e., dominant follicle diameter of 1.8–2.0 cm), 10,000 IU of human chorionic gonadotropin (hCG) is administered intramuscularly to trigger ovulation, which typically occurs within 48 h post-injection. Following ovulation, oral progesterone capsules will be administered at a dose of 10 mg twice daily for luteal phase support. Treatment will continue until the serum β-hCG level is performed. If the pregnancy test is positive, dydrogesterone will be continued until clinical pregnancy is confirmed by ultrasonography. β-hCG levels will be measured 14 days later to confirm pregnancy. In cases of positive results, follow-up transvaginal ultrasonography will be performed 14 days thereafter to confirm clinical pregnancy. If the pregnancy test is negative, OI may be repeated in the next cycle, provided that normal baseline ultrasonographic findings are confirmed on days 3–5 of the subsequent menstrual cycle.

All participants will be strongly recommended to undergo hysterosalpingography (HSG) prior to treatment initiation to confirm bilateral tubal patency and to minimize potential bias between study groups. The decision to perform HSG will ultimately be made by the attending physician in consultation with the participant, considering individual clinical circumstances. Participants who fail to achieve pregnancy after 5 cycles should be referred for assisted reproductive technologies.

#### Safety monitoring

Adverse events will be actively monitored and documented throughout the 12-week intervention period in both treatment groups. In the metformin group, particular attention will be paid to gastrointestinal adverse effects, including nausea, vomiting, abdominal discomfort, and diarrhea. Participants who experience metformin intolerance will undergo clinical evaluation, and dose adjustment or discontinuation will be considered when necessary. In the CR diet group, potential diet-related adverse events or symptoms associated with energy restriction will be monitored by investigators and dietitians during follow-up, and the dietary intervention will be adjusted when clinically indicated. All adverse events will be documented with respect to their nature, severity, timing, management, outcome, and assessed relationship to the study intervention. Serious adverse events will be promptly evaluated by the principal investigator in collaboration with the Data Monitoring Committee (DMC), and appropriate clinical management and reporting procedures will be undertaken in accordance with the study protocol and applicable ethical requirements. The DMC will review relevant safety information and may recommend modification or discontinuation of the intervention or trial when warranted.

#### Outcomes and definitions

The primary outcome is live birth, defined as the delivery of one or more living infants at ≥22 completed weeks of gestation or with a birth weight >500 g. Live birth will be confirmed and recorded based on hospital medical records and clinical documentation. For multiple pregnancies, the delivery of one or more live-born infants from the same pregnancy will be counted as one live birth event at the participant level for the primary outcome, while the number of live-born infants and plurality will be recorded separately. Pregnancy losses, including miscarriage and stillbirth, will be recorded separately and will not be classified as live birth events.

#### Data collection and management

**Figure 2:**
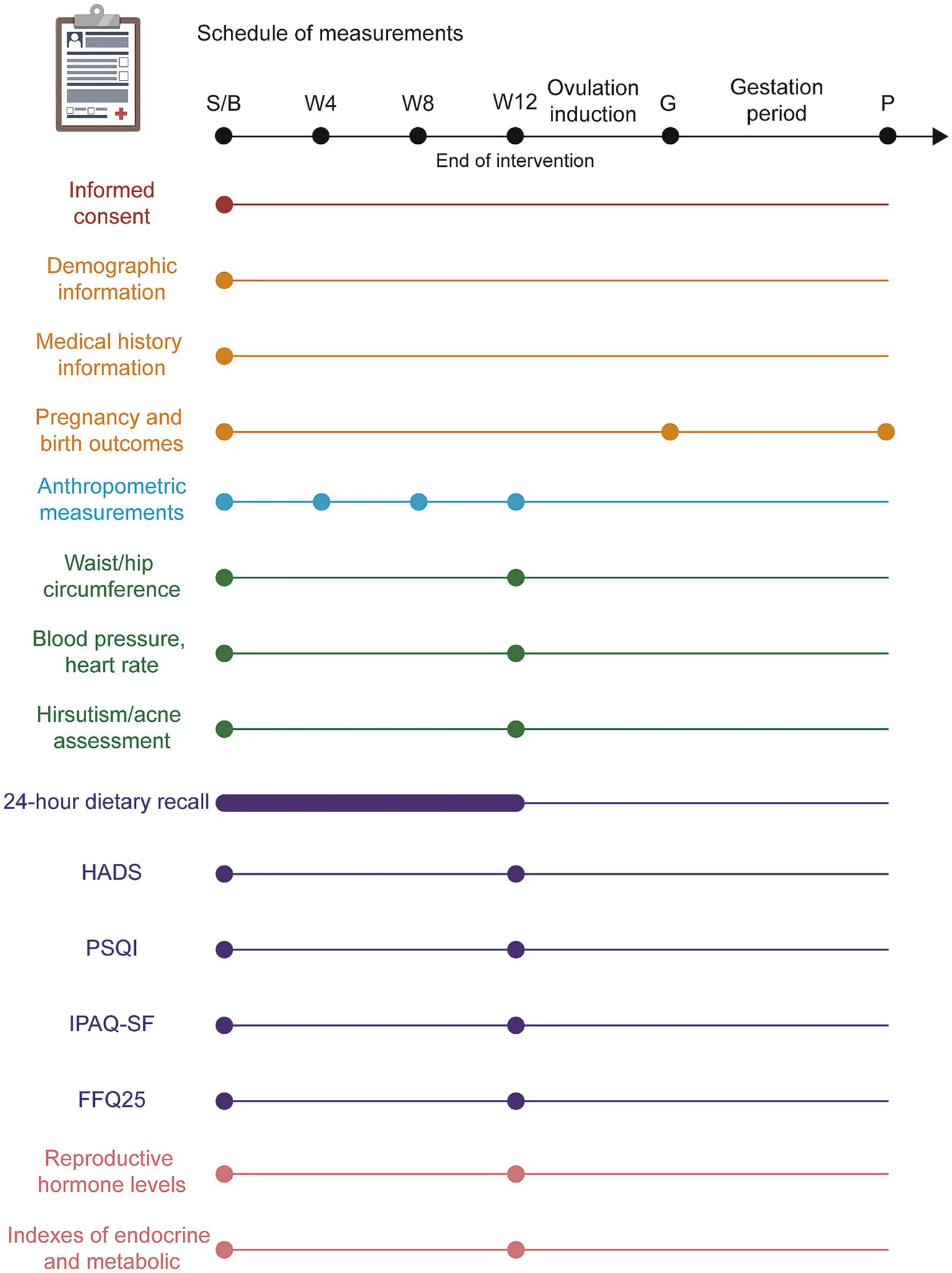
Schedule of measurements. S/B, Screening/baseline; W 4, Week 4; W 8, Week 8; W 12, Week 12; G, Gestation; P, Parturition; HADS, Hospital Anxiety and Depression Scale; PSQI, Pittsburgh Sleep Quality Index; IPAQ-SF, International Physical Activity Questionnaire Short Form; FFQ, Food Frequency Questionnaire.

Figure 2 summarizes the measurements collected at each scheduled visit.

#### Demographic information

Data will be collected on participants’ age, ethnicity, marital status, educational background, menstrual history, reproductive history, living environment, lifestyle factors, employment status, smoking status, alcohol consumption, and relevant characteristics of their spouses.

#### Physical measurements

Body weight and composition will be assessed using an InBody analyzer or body fat scales. Waist circumference will be measured once at the midpoint between the lowest rib and the iliac crest, while hip circumference will be measured once at the widest part of the buttocks. Blood pressure (BP) will be measured once with the participant seated and at rest for at least 10 min.

#### Questionnaires

The modified Ferriman–Gallwey score (mF-G score) of 4 will be used to detect hirsutism (14). The Hospital Anxiety and Depression Scale (HADS) is a widely used self-report questionnaire for assessing emotional distress, specifically anxiety and depression (56). It consists of 14 items, with seven assessing anxiety and seven assessing depression. Each item is rated on a 4-point scale ranging from 0 to 3. A total score above 8 suggests mild symptoms, while a score above 10 indicates moderate symptoms (57). The Pittsburgh Sleep Quality Index (PSQI) is a commonly used self-report measure of sleep quality and disturbances over a one-month period. The global PSQI score ranges from 0 to 21, with higher scores indicating worse sleep quality; a score ≥5 indicates poor sleep quality (58). Physical activity will be assessed using six items from IPAQ-SF, which captures the frequency and duration of vigorous activity, moderate activity, and walking. Data are used to classify participants into high, moderate, or low physical activity groups. Time spent in each activity category is weighted by energy expenditure to calculate MET-minutes per week (51, 52). Dietary intake over the past 12 months will be assessed at baseline using a validated semi-quantitative 25-item food frequency questionnaire (FFQ-25), which has been validated for evaluating habitual dietary consumption in Chinese populations (59–61). Participants will be asked to report the frequency (daily, weekly, monthly, yearly, or never) and quantity (using the standard Chinese unit liang, where 1 liang = 50 g) of consumption for each food item over the past 12 months (62). To objectively assess changes in dietary intake during the 12-week intervention period, three 24-h dietary recalls (covering two weekdays and one weekend day) will be extracted by dietitians from uploaded app-based food diary records of participants. Daily dietary intake will be calculated based on the reported food types and portion sizes, using the nutrient composition data from the Chinese Food Composition Table (55).

#### Indexes of endocrine and metabolic function

Fasting plasma glucose, fasting insulin, 75 g oral glucose tolerance test (OGTT), insulin release test (IRT), liver and kidney function, blood lipids, thyroid function, and 25-hydroxyvitamin D will be assessed. For the OGTT and IRT, blood samples will be collected at 0 (fasting) and 2 h after glucose ingestion to assess glucose and insulin responses. Glucose concentrations will be measured using a glucose oxidase–based enzymatic assay. Serum lipid profiles, including total cholesterol and triglycerides, will be quantified via standard enzymatic colorimetric methods. Serum insulin concentrations and thyroid function parameters—thyrotropin, free triiodothyronine, and free thyroxine—will be determined using validated immunoassays. Liver function parameters, including alanine aminotransferase and aspartate aminotransferase, as well as serum uric acid, will also be analyzed using enzymatic methods. 25-hydroxyvitamin D concentrations will be assessed by mass spectrometry.

#### Sex hormone levels

LH, follicle-stimulating hormone, prolactin, T, estradiol, progesterone, 17α-hydroxyprogesterone, SHBG, and FAI will be measured using validated immunoassays, at both baseline and post-intervention.

The data sources for this study will consist of medical records and follow-up documentation, which will be systematically entered and securely maintained using an electronic data capture (EDC) system. Outcome ascertainment will be performed by assessors blinded to treatment allocation using standardized medical records and follow-up documentation. To ensure data integrity and accuracy, a three-tiered quality control framework will be implemented. Firstly, the EDC system will integrate automated real-time validation checks to evaluate logical consistency and completeness of the entered data. Secondly, clinical researchers will perform source data verification, while trained data entry personnel will be responsible for accurate data input and timely updates, thereby minimizing human error. Thirdly, an independent Data Monitoring Committee (DMC) consisting of clinical, statistical, and ethical experts will conduct periodic audits to oversee data quality and compliance with study protocols. Any identified discrepancies will be promptly flagged within the system, and the respective data entry staff will be notified to investigate and rectify these inconsistencies in a timely manner.

#### Statistical analysis

The primary analysis will follow the intention-to-treat principle, with all randomized participants analyzed according to their originally assigned treatment group regardless of intervention adherence or protocol deviations. For continuous baseline characteristics, normality will be assessed using frequency histograms and the Shapiro–Wilk test. If normally distributed, data will be presented as mean ± standard deviation (SD); otherwise, median and interquartile ranges (IQRs) will be reported. Student’s t-test or the Wilcoxon rank-sum test will be used to compare differences between groups, as appropriate. For categorical variables, frequencies and percentages will be reported for both groups. Additionally, the number of recruited participants, those lost to follow-up, protocol violations, and other relevant descriptive statistics will be documented.

The primary outcome, live birth rate, will be compared between the two groups using Pearson’s chi-square test or Fisher’s exact test for unadjusted analyses. The unadjusted risk ratio (RR) and 95% confidence interval (CI) will be estimated. In addition, an adjusted analysis will be performed using multivariable Poisson regression or a log-binomial model, as appropriate, to estimate the adjusted RR and 95% CI. Prespecified clinically relevant baseline prognostic factors that are routinely available across the study population, including age, baseline body mass index (BMI), and relevant baseline PCOS-related reproductive and metabolic characteristics, will be considered in the adjusted model. Hazard ratios (HR) and 95% CI will be calculated using multivariable Cox proportional hazards analysis to compare time to live birth between groups, with adjustment for relevant prespecified baseline prognostic factors. Pregnancy rates will also be assessed using an intention-to-treat approach with Kaplan–Meier estimation, where time to pregnancy is defined as the number of days from randomization (intervention start) to the first positive pregnancy test. Between-group differences will be evaluated using the log-rank test, and multivariable Cox proportional hazards models will be applied to adjust for the prespecified baseline prognostic factors. As anti-Müllerian hormone (AMH) measurements are not available for all participants, AMH will not be included as a covariate in the primary adjusted analysis. Among participants with available AMH measurements, supplementary exploratory analyses will be conducted to assess the association between baseline AMH and reproductive outcomes and to explore whether baseline AMH modifies the relative effects of the CR diet and metformin.

For the primary outcome, participants who are lost to follow-up and whose live birth status cannot be ascertained will be conservatively classified as not having achieved a live birth. The number and reasons for withdrawal and loss to follow-up will be documented and reported separately for each treatment group. Missing baseline covariate data will be handled using complete-case analysis in the corresponding adjusted models. Multiple imputation will be used in sensitivity analyses to assess the robustness of the results to missing covariate data. Additional sensitivity analyses will be performed to evaluate the potential impact of missing outcome data and loss to follow-up on the primary findings. These analyses will examine alternative assumptions regarding outcomes among participants lost to follow-up and will be compared with the primary analysis in which missing live birth outcomes are classified as no live birth.

Participants with insufficient intervention adherence or protocol deviations will remain included in the primary intention-to-treat analysis according to their randomized treatment assignment. Additional sensitivity analyses will be conducted to assess the potential impact of major protocol deviations on the primary findings. A per-protocol analysis will be conducted as a supportive secondary analysis and will include participants who complete the trial, meet the prespecified intervention-specific adherence criteria, and have no major protocol violations. Primary and secondary outcomes will be compared between treatment groups within the per-protocol population. The per-protocol findings will be interpreted as supportive analyses alongside the primary intention-to-treat results. Secondary outcomes will be compared between groups using a similar approach for categorical variables. Mixed-effects models will be applied for repeated measurements. Exploratory subgroup analyses will be conducted according to prespecified baseline characteristics, including age and BMI. Potential heterogeneity in intervention effects across these characteristics will be assessed by including treatment-by-subgroup interaction terms in the corresponding regression models. Where AMH measurements are available, the potential effect-modifying role of AMH will also be explored in supplementary analyses.

All statistical analyses will be performed using SAS version 9.4 (SAS Institute Inc., Cary, NC, USA). All tests will be two-tailed, and a *p*-value < 0.05 will be considered statistically significant.

## Discussion

This trial investigates the comparative efficacy of a 12-week CR diet versus metformin administered prior to OI in women with PCOS and overweight/obesity, with live birth as the primary outcome. Metformin represents an established pharmacological approach targeting insulin resistance and related endocrine abnormalities in PCOS, whereas CR diet provides a non-pharmacological approach targeting overlapping metabolic disturbances through energy restriction and weight loss. The direct comparison of these strategies will therefore address whether preconception metabolic optimization through CR diet can achieve superior reproductive outcomes compared with metformin. Beyond the primary superiority hypothesis, this comparison may also help clarify the potential role of CR diet as a non-pharmacological strategy for metabolic and reproductive optimization before OI.

Reproductive outcomes in women with PCOS may be influenced not only by metabolic and endocrine abnormalities but also by individual baseline reproductive characteristics. Age, BMI, and ovarian reserve-related markers such as AMH may contribute to heterogeneity in reproductive prognosis. Recent observational studies have highlighted the potential relevance of age, BMI, and AMH to reproductive prognosis in women with PCOS undergoing fertility treatment, including artificial insemination and *in vitro* fertilization (38, 39). In the present trial, routinely available baseline prognostic factors, including age and BMI, will be considered in adjusted analyses. As AMH measurements are not available for all participants, AMH will be evaluated as an exploratory prognostic factor in the subset of participants with available data. These analyses may provide additional insight into individual variability in reproductive prognosis and treatment response.

Previous intervention studies on CR diets in PCOS have not directly compared CR diets with metformin, and existing evidence remains limited (63). The present study systematically evaluates the effects of these two interventions on reproductive, metabolic, and endocrine outcomes. The use of an app-based CR diet intervention enables remote dietary monitoring and facilitates implementation across geographically diverse study sites. The use of an app-based CR diet intervention enables remote dietary monitoring and facilitates implementation across geographically diverse study sites. This approach may be particularly beneficial for participants in rural or remote areas where access to PCOS-related healthcare services may be limited. In addition, the application facilitates participant engagement through systematic dietary monitoring and regular feedback from dietitians, which may enhance adherence to the intervention and support behavioral changes. Within the prescribed energy and macronutrient targets, participants retain flexibility in food selection according to their usual dietary habits and preferences, which may also improve the feasibility of implementing the CR intervention across different settings.

The duration and timing of preconception interventions may influence subsequent metabolic, endocrine, and reproductive outcomes. The 12-week intervention period used in the present trial was selected to provide a feasible window for implementing CR diet or metformin before OI while minimizing unnecessary delays in fertility treatment. However, this trial is not designed to directly compare different intervention durations, and therefore the optimal duration of preconception treatment cannot be determined. A shorter intervention period may provide insufficient time to achieve meaningful metabolic or endocrine changes, whereas a longer intervention may allow greater or more sustained improvements but may also delay the initiation of fertility treatment and potentially affect adherence. Future studies directly comparing different intervention durations and the timing of transition to OI are warranted to determine the optimal preconception treatment strategy for women with PCOS and overweight/obesity.

Several limitations of this study should be acknowledged. First, because of the distinct nature of the interventions, participants and personnel delivering the interventions cannot be blinded to treatment allocation, which may introduce performance bias. Nevertheless, outcome assessors and data analysts will remain blinded, and live birth, the primary outcome, is an objective clinical endpoint, which may reduce the potential for assessment bias. Second, the intensity of intervention differs between the two groups. Participants in the CR diet group receive app-based dietary monitoring, individualized dietary guidance, and regular feedback from dietitians, whereas participants in the metformin group receive pharmacological treatment with routine clinical advice. Consequently, observed between-group differences may partly reflect the additional healthcare attention, self-monitoring, and behavioral support provided as components of the CR intervention rather than calorie restriction alone. Attention bias therefore cannot be completely excluded, and the findings should be interpreted as reflecting the comparative effectiveness of the overall CR diet and metformin intervention strategies rather than the isolated physiological effects of calorie restriction versus metformin. Third, although standardized study procedures are used to promote consistency across participating centers, some center-level variability in intervention delivery and measurements cannot be completely excluded. In particular, differences in body composition assessment devices across centers may introduce measurement variability in anthropometric outcomes. Finally, the study population is restricted to women aged 20–35 years with PCOS and overweight/obesity who are eligible for OI, which may limit the generalizability of the findings to women outside this age range, women with PCOS without overweight/obesity, or those receiving other forms of fertility treatment. In addition, because the trial is conducted in China and applies an Asian-specific BMI threshold, extrapolation of the findings to populations with different ethnic, dietary, and healthcare backgrounds should be made cautiously.

This trial will compare the efficacy of a CR diet and metformin intervention in women with PCOS and overweight/obese, with live birth rate as the primary outcome. The findings will provide valuable insights into the comparative effectiveness of CR diet versus metformin therapy and the potential utility of CR diet as a broader therapeutic strategy for managing women with PCOS and overweight/obesity. Furthermore, this investigation will generate novel evidence to support the development of future app-based dietary interventions, potentially improving access to care for overweight/obese PCOS populations in China and beyond.

## Glossary

App: Application
BMI: Body mass index
BMR: Basal metabolic rate
β-hCG: β-human chorionic gonadotropin
CI: Confidence interval
CONSORT: Consolidated Standards of Reporting Trials
CR: Calorie-restricted
DMC: Data Monitoring Committee
EDC: Electronic data capture
FAI: Free androgen index
FFQ-25: 25-item food frequency questionnaire
HADS: Hospital Anxiety and Depression Scale
HMG: Human menopausal gonadotropin
HR: Hazard ratios
HSG: Hysterosalpingography
hCG: Human chorionic gonadotropin
IPAQ-SF: International Physical Activity Questionnaire Short Form
IRT: Insulin release test
LH: Luteinizing hormone
mF-G score: Modified Ferriman–Gallwey score
OGTT: Oral glucose tolerance test
OI: Ovulation induction
PAL: Physical activity level
PCOS: Polycystic ovary syndrome
PSQI: Pittsburgh Sleep Quality Index
RR: Risk ratio
SD: Standard deviation
SHBG: Sex hormone-binding globulin
SPIRIT: Standard Protocol Items Recommendations for Interventional Trials
T: Testosterone
TEE: Total energy expenditure

## References

[1] Melin J Forslund M Alesi S Piltonen T Romualdi D Spritzer PM . Metformin and combined Oral contraceptive pills in the Management of Polycystic Ovary Syndrome: a systematic review and Meta-analysis. J Clin Endocrinol Metab. (2024) 109:e817–36. doi: 10.1210/clinem/dgad465, 37554096 PMC10795934

[2] Teede HJ Tay CT Laven JJE Dokras A Moran LJ Piltonen TT . Recommendations from the 2023 international evidence-based guideline for the assessment and management of polycystic ovary syndrome. J Clin Endocrinol Metab. (2023) 108:2447–69. doi: 10.1210/clinem/dgad463, 37580314 PMC10505534

[3] Stener-Victorin E Teede H Norman RJ Legro R Goodarzi MO Dokras A . Polycystic ovary syndrome. Nat Rev Dis Primers. (2024) 10:27. doi: 10.1038/s41572-024-00511-3, 38637590

[4] Safiri S Noori M Nejadghaderi SA Karamzad N Carson-Chahhoud K Sullman MJM . Prevalence, incidence and years lived with disability due to polycystic ovary syndrome in 204 countries and territories, 1990-2019. Hum Reprod. (2022) 37:1919–31. doi: 10.1093/humrep/deac091, 35586937

[5] Goodarzi MO Dumesic DA Chazenbalk G Azziz R. Polycystic ovary syndrome: etiology, pathogenesis and diagnosis. Nat Rev Endocrinol. (2011) 7:219–31. doi: 10.1038/nrendo.2010.217, 21263450

[6] Walter K. What is polycystic ovary syndrome? JAMA. (2022) 327:294. doi: 10.1001/jama.2021.19776, 35040885

[7] Sortino MA Salomone S Carruba MO Drago F. Polycystic ovary syndrome: insights into the therapeutic approach with Inositols. Front Pharmacol. (2017) 8:341. doi: 10.3389/fphar.2017.00341, 28642705 PMC5463048

[8] Liu X Zhang J Wang S. Global, regional, and national burden of infertility attributable to PCOS, 1990-2019. Hum Reprod. (2023) 39:108–18. doi: 10.1093/humrep/dead241 38011904

[9] Lonardo MS Cacciapuoti N Guida B Di Lorenzo M Chiurazzi M Damiano S . Hypothalamic-ovarian axis and adiposity relationship in polycystic ovary syndrome: physiopathology and therapeutic options for the Management of Metabolic and Inflammatory Aspects. Curr Obes Rep. (2024) 13:51–70. doi: 10.1007/s13679-023-00531-2, 38172476 PMC10933167

[10] Myers SH Russo M Dinicola S Forte G Unfer V. Questioning PCOS phenotypes for reclassification and tailored therapy. Trends Endocrinol Metab. (2023) 34:694–703. doi: 10.1016/j.tem.2023.08.005, 37661546

[11] Glueck CJ Goldenberg N. Characteristics of obesity in polycystic ovary syndrome: etiology, treatment, and genetics. Metabolism. (2019) 92:108–20. doi: 10.1016/j.metabol.2018.11.002, 30445140

[12] Góral A Żywot K Zalewski W Jagodziński A Murawski M. Polycystic ovary syndrome and eating disorders-a literature review. J Clin Med. (2024) 14:27. doi: 10.3390/jcm14010027, 39797110 PMC11720544

[13] Fan H Ren Q Sheng Z Deng G Li L. The role of the thyroid in polycystic ovary syndrome. Front Endocrinol. (2023) 14:1242050. doi: 10.3389/fendo.2023.1242050, 37867519 PMC10585146

[14] Teede HJ Tay CT Laven JJE Dokras A Moran LJ Piltonen TT . Recommendations from the 2023 international evidence-based guideline for the assessment and management of polycystic ovary syndrome. Eur J Endocrinol. (2023) 189:G43–g64. doi: 10.1093/ejendo/lvad096 37580861

[15] Viollet B Guigas B Sanz Garcia N Leclerc J Foretz M Andreelli F. Cellular and molecular mechanisms of metformin: an overview. Clin Sci. (2012) 122:253–70. doi: 10.1042/cs20110386, 22117616 PMC3398862

[16] Tso LO Costello MF Albuquerque LET Andriolo RB Macedo CR. Metformin treatment before and during IVF or ICSI in women with polycystic ovary syndrome. Cochrane Database Syst Rev. (2020) 12:Cd006105. doi: 10.1002/14651858.CD006105.pub4 PMC8171384 33347618

[17] Morin-Papunen L Rantala AS Unkila-Kallio L Tiitinen A Hippeläinen M Perheentupa A . Metformin improves pregnancy and live-birth rates in women with polycystic ovary syndrome (PCOS): a multicenter, double-blind, placebo-controlled randomized trial. J Clin Endocrinol Metab. (2012) 97:1492–500. doi: 10.1210/jc.2011-3061, 22419702

[18] Guan HR Li B Zhang ZH Wu HS Wang N Chen XF . Exploring the efficacy and mechanism of bailing capsule to improve polycystic ovary syndrome in mice based on intestinal-derived LPS-TLR4 pathway. J Ethnopharmacol. (2024) 331:118274. doi: 10.1016/j.jep.2024.118274, 38697410

[19] Kupreeva M Diane A Lehner R Watts R Ghosh M Proctor S . Effect of metformin and flutamide on insulin, lipogenic and androgen-estrogen signaling, and cardiometabolic risk in a PCOS-prone metabolic syndrome rodent model. Am J Physiol Endocrinol Metab. (2019) 316:E16–e33. doi: 10.1152/ajpendo.00018.2018, 30153063 PMC6417686

[20] Aubuchon M Lieman H Stein D Cohen HW Isaac B Adel G . Metformin does not improve the reproductive or metabolic profile in women with polycystic ovary syndrome (PCOS). Reproduct Sci. (2009) 16:938–46. doi: 10.1177/1933719109340925, 19692630

[21] Sturrock ND Lannon B Fay TN. Metformin does not enhance ovulation induction in clomiphene resistant polycystic ovary syndrome in clinical practice. Br J Clin Pharmacol. (2002) 53:469–73. doi: 10.1046/j.1365-2125.2002.01575.x, 11994052 PMC1874363

[22] McCreight LJ Bailey CJ Pearson ER. Metformin and the gastrointestinal tract. Diabetologia. (2016) 59:426–35. doi: 10.1007/s00125-015-3844-9, 26780750 PMC4742508

[23] Sánchez-Garrido MA Serrano-López V Ruiz-Pino F Vázquez MJ Rodríguez-Martín A Torres E . Superior metabolic improvement of polycystic ovary syndrome traits after GLP1-based multi-agonist therapy. Nat Commun. (2024) 15:8498. doi: 10.1038/s41467-024-52898-y, 39353946 PMC11445520

[24] Shang Y Zhou H Hu M Feng H. Effect of diet on insulin resistance in polycystic ovary syndrome. J Clin Endocrinol Metab. (2020) 105:dgaa425. doi: 10.1210/clinem/dgaa425, 32621748

[25] Juhász AE Stubnya MP Teutsch B Gede N Hegyi P Nyirády P . Ranking the dietary interventions by their effectiveness in the management of polycystic ovary syndrome: a systematic review and network meta-analysis. Reprod Health. (2024) 21:28. doi: 10.1186/s12978-024-01758-5, 38388374 PMC10885527

[26] Shang Y Zhou H He R Lu W. Dietary modification for reproductive health in women with polycystic ovary syndrome: a systematic review and meta-analysis. Front Endocrinol. (2021) 12:735954. doi: 10.3389/fendo.2021.735954, 34790167 PMC8591222

[27] Yang J Liang J Xu T Lin Q Ye Q Lin F . The impact of dietary interventions on polycystic ovary syndrome patients with a BMI ≥25 kg/m(2): a systematic review and meta-analysis of randomized controlled trials. Reprod Med Biol. (2024) 23:e12607. doi: 10.1002/rmb2.12607 PMC11442045 39351128

[28] Lim SS Hutchison SK Van Ryswyk E Norman RJ Teede HJ Morna LJ. Lifestyle changes in women with polycystic ovary syndrome. Cochrane Database Syst Rev. (2019) 3:Cd007506. 10.1002/14651858.CD007506.pub4 PMC6438659 30921477

[29] McGowan M Garad R Wadhwani G Torkel S Rao V Maunder A . Understanding barriers and facilitators to lifestyle management in people with polycystic ovary syndrome: a mixed method systematic review. Obes Rev. (2025) 26:e13854. doi: 10.1111/obr.13854, 39462252

[30] Torkel S Wang R Norman RJ Zhao L Liu K Boden D . Barriers and enablers to a healthy lifestyle in people with infertility: a mixed-methods systematic review. Hum Reprod Update. (2024) 30:569–83. doi: 10.1093/humupd/dmae011, 38743500 PMC11369225

[31] Torkel S Moran L Wang R Villani A Mantziorkel E Norman RJ . Barriers and enablers to a healthy lifestyle in people with infertility: a qualitative descriptive study. Reproduct Biol Endocrinol. (2025) 23:52. doi: 10.1186/s12958-025-01387-y PMC11974000 40197283

[32] Rosen CJ Ingelfinger JR. GLP-1 receptor agonists. N Engl J Med. (2026) 394:1313–24. doi: 10.1056/nejmra2500106, 41931049

[33] Cena H Chiovato L Nappi RE. Obesity, polycystic ovary syndrome, and infertility: a new avenue for GLP-1 receptor agonists. J Clin Endocrinol Metab. (2020) 105:e2695–709. doi: 10.1210/clinem/dgaa285, 32442310 PMC7457958

[34] Most J Redman LM. Impact of calorie restriction on energy metabolism in humans. Exp Gerontol. (2020) 133:110875. doi: 10.1016/j.exger.2020.110875, 32057825 PMC9036397

[35] Holte J Bergh T Berne C Wide L Lithell H. Restored insulin sensitivity but persistently increased early insulin secretion after weight loss in obese women with polycystic ovary syndrome. J Clin Endocrinol Metab. (1995) 80:2586–93. doi: 10.1210/jcem.80.9.7673399, 7673399

[36] Andersen P Seljeflot I Abdelnoor M Arnesen H Dale PO Løvik A . Increased insulin sensitivity and fibrinolytic capacity after dietary intervention in obese women with polycystic ovary syndrome. Metab Clin Exp. (1995) 44:611–6. doi: 10.1016/0026-0495(95)90118-3, 7752909

[37] Esfahanian F Zamani MM Heshmat R Moini nia F. Effect of metformin compared with hypocaloric diet on serum C-reactive protein level and insulin resistance in obese and overweight women with polycystic ovary syndrome. J Obstet Gynaecol Res. (2013) 39:806–13. doi: 10.1111/j.1447-0756.2012.02051.x, 23279603

[38] Moreira T Leal C Barreiro M Tomé A Vale-Fernandes E. Predictors of pregnancy after artificial insemination in women with polycystic ovary syndrome. JBRA Assist Reprod. (2025) 29:201–10. doi: 10.5935/1518-0557.20240095 PMC12225176 39983029

[39] Ribeiro MP Lemos C Leal C Barreiro M Tomé A Vale-Fernandes E. Reproductive prognosis factors in women with infertility and polycystic ovary syndrome undergoing in vitro fertilization techniques. JBRA Assist Reprod. (2025) 29:583–600. doi: 10.5935/1518-0557.20250040 PMC12694962 40794535

[40] Kiyosue A Dunn JP Cui X Hickey A Hirase T Imaoka T . Safety and efficacy analyses across age and body mass index subgroups in east Asian participants with type 2 diabetes in the phase 3 tirzepatide studies (SURPASS programme). Diabetes Obes Metab. (2023) 25:1056–67. doi: 10.1111/dom.14952, 36545807

[41] Arya S Hansen KR Peck JD Wild RA H. National Institute of Child, and Development Reproductive MedicineN. Human. Metabolic syndrome in obesity: treatment success and adverse pregnancy outcomes with ovulation induction in polycystic ovary syndrome. Am J Obstet Gynecol. (2021) 225:280 e1–280 e11. doi: 10.1016/j.ajog.2021.03.048 PMC8429086 33852887

[42] Legro RS Brzyski RG Diamond MP Coutifaris C Schlaff WD Casson P . Letrozole versus clomiphene for infertility in the polycystic ovary syndrome. N Engl J Med. (2014) 371:119–29. doi: 10.1056/NEJMoa1313517, 25006718 PMC4175743

[43] Franchignoni F Vercelli S Giordano A Sartorio F Bravini E Ferriero G. Minimal clinically important difference of the disabilities of the arm, shoulder and hand outcome measure (DASH) and its shortened version (QuickDASH). J Orthop Sports Phys Ther. (2014) 44:30–9. doi: 10.2519/jospt.2014.4893, 24175606

[44] Zhang Y Qu Z Lu T Shao X Cai M Dilimulati D . Effects of a Dulaglutide plus calorie-restricted diet versus a calorie-restricted diet on visceral fat and metabolic profiles in women with polycystic ovary syndrome: a randomized controlled trial. Nutrients. (2023) 15:556. doi: 10.3390/nu15030556, 36771262 PMC9920202

[45] Sun J Ruan Y Xu N Wu P Lin N Yuan K . The effect of dietary carbohydrate and calorie restriction on weight and metabolic health in overweight/obese individuals: a multi-center randomized controlled trial. BMC Med. (2023) 21:192. doi: 10.1186/s12916-023-02869-9 PMC10210464 37226271

[46] Lin S Cienfuegos S Ezpeleta M Gabel K Pavlou V Mulas A . Time-restricted eating without calorie counting for weight loss in a racially diverse population: a randomized controlled trial. Ann Intern Med. (2023) 176:885–95. doi: 10.7326/M23-0052, 37364268 PMC11192144

[47] Barnosky AR Hoddy KK Unterman TG Varady KA. Intermittent fasting vs daily calorie restriction for type 2 diabetes prevention: a review of human findings. Transl Res. (2014) 164:302–11. doi: 10.1016/j.trsl.2014.05.013, 24993615

[48] Millward DJ. A new approach to establishing dietary energy reference values. Curr Opin Clin Nutr Metab Care. (2012) 15:413–7. doi: 10.1097/MCO.0b013e3283562c26, 22878235

[49] Shetty P. Energy requirements of adults. Public Health Nutr. (2005) 8:994–1009. doi: 10.1079/phn2005792, 16277816

[50] Ryan AS Novitskaya M Treuth AL. Predictive equations overestimate resting metabolic rate in survivors of chronic stroke. Arch Phys Med Rehabil. (2022) 103:1352–9. doi: 10.1016/j.apmr.2022.01.155, 35192798 PMC9246861

[51] Bauman A Ainsworth BE Sallis JF Hagströmer M Craig CL Bull FC . The descriptive epidemiology of sitting. A 20-country comparison using the international physical activity questionnaire (IPAQ). Am J Prev Med. (2011) 41:228–35. doi: 10.1016/j.amepre.2011.05.003, 21767731

[52] Duncan MJ Arbour-Nicitopoulos K Subramaniapillai M Remington G Faulkner G. Revisiting the international physical activity questionnaire (IPAQ): assessing sitting time among individuals with schizophrenia. Psychiatry Res. (2019) 271:311–8. doi: 10.1016/j.psychres.2018.11.063, 30529312

[53] Plucker A Thomas DM Broskey N Martin CK Schoeller D Shook R . Adult energy requirements predicted from doubly labeled water. Int J Obes. (2005) 42:1515–23. doi: 10.1038/s41366-018-0168-0 PMC9218950 30026590

[54] U.S.D.o.A.U.S.D.o.H.a.H. Services. Dietary Guidelines for Americans, 2020–2025. 9th ed. Washington, DC: U.S. Department of Agriculture (2020).

[55] Yang Y. China Food Composition Tables Standard Edition (Six Edition). Beijing: Peking University Medical Press (2018).

[56] Annunziata MA Muzzatti B Bidoli E Flaiban C Bomben F Piccinin M . Hospital anxiety and depression scale (HADS) accuracy in cancer patients. Support Care Cancer. (2020) 28:3921–6. doi: 10.1007/s00520-019-05244-8, 31858249

[57] Cassiani-Miranda CA Scoppetta O Cabanzo-Arenas DF. Validity of the hospital anxiety and depression scale (HADS) in primary care patients in Colombia. Gen Hosp Psychiatry. (2022) 74:102–9. doi: 10.1016/j.genhosppsych.2021.01.014, 33750606

[58] Watson EJ Coates AM Kohler M Banks S. Caffeine consumption and sleep quality in Australian adults. Nutrients. (2016) 8:479. doi: 10.3390/nu8080479, 27527212 PMC4997392

[59] El Kinany K Garcia-Larsen V Khalis M Deoula MMS Benslimane A Ibrahim A . Adaptation and validation of a food frequency questionnaire (FFQ) to assess dietary intake in Moroccan adults. Nutr J. (2018) 17:61. doi: 10.1186/s12937-018-0368-4, 29895304 PMC5998554

[60] Iwasaki Y Sakamoto M Nakai K Oka T Dakeishi M Iwata T . Estimation of daily mercury intake from seafood in Japanese women: Akita cross-sectional study. Tohoku J Exp Med. (2003) 200:67–73. doi: 10.1620/tjem.200.67, 12962403

[61] Jiang X Wang X Zhang M Yu L He J Wu S . Associations between specific dietary patterns, gut microbiome composition, and incident subthreshold depression in Chinese young adults. J Adv Res. (2024) 65:183–95. doi: 10.1016/j.jare.2024.05.030, 38879123 PMC11518947

[62] Ye Q Hong X Wang Z Yang H Chen X Zhou H . Reproducibility and validity of an FFQ developed for adults in Nanjing, China. Br J Nutr. (2016) 115:887–94. doi: 10.1017/S0007114515005334, 26785928

[63] García-Gómez E Gómez-Viais YI Cruz-Aranda MM Martínez-Razo LD Reyes-Mayoral C Ibarra-González L . The effect of metformin and carbohydrate-controlled diet on DNA methylation and gene expression in the endometrium of women with polycystic ovary syndrome. Int J Mol Sci. (2023) 24:6857. doi: 10.3390/ijms24076857, 37047828 PMC10094785

